# Modeling Social Sensory Processing During Social Computerized Cognitive Training for Psychosis Spectrum: The Resting-State Approach

**DOI:** 10.3389/fpsyt.2020.554475

**Published:** 2020-11-19

**Authors:** Lana Kambeitz-Ilankovic, Julian Wenzel, Shalaila S. Haas, Anne Ruef, Linda A. Antonucci, Rachele Sanfelici, Marco Paolini, Nikolaos Koutsouleris, Bruno Biagianti

**Affiliations:** ^1^Department of Psychiatry, Faculty of Medicine and University Hospital of Cologne, University of Cologne, Cologne, Germany; ^2^Department of Psychiatry and Psychotherapy, Ludwig-Maximilian University, Munich, Germany; ^3^Department of Psychiatry, Icahn School of Medicine at Mount Sinai, New York, NY, United States; ^4^Department of Education, Psychology, Communication, University of Bari “Aldo Moro”, Bari, Italy; ^5^Max Planck School of Cognition, Leipzig, Germany; ^6^Department of Radiology, University Hospital, Ludwig-Maximilian-University, Munich, Germany; ^7^Department of Pathophysiology and Transplantation, University of Milan, Milan, Italy; ^8^Department of R&D, Posit Science Corporation, San Francisco, CA, United States

**Keywords:** computerized cognitive training, sensory processing, social cognition, resting state, functional connectivity

## Abstract

**Background:** Greater impairments in early sensory processing predict response to auditory computerized cognitive training (CCT) in patients with recent-onset psychosis (ROP). Little is known about neuroimaging predictors of response to social CCT, an experimental treatment that was recently shown to induce cognitive improvements in patients with psychosis. Here, we investigated whether ROP patients show interindividual differences in sensory processing change and whether different patterns of SPC are (1) related to the differential response to treatment, as indexed by gains in social cognitive neuropsychological tests and (2) associated with unique resting-state functional connectivity (rsFC).

**Methods:** Twenty-six ROP patients completed 10 h of CCT over the period of 4–6 weeks. Subject-specific improvement in one CCT exercise targeting early sensory processing—a speeded facial Emotion Matching Task (EMT)—was studied as potential proxy for target engagement. Based on the median split of SPC from the EMT, two patient groups were created. Resting-state activity was collected at baseline, and bold time series were extracted from two major default mode network (DMN) hubs: left medial prefrontal cortex (mPFC) and left posterior cingulate cortex (PCC). Seed rsFC analysis was performed using standardized Pearson correlation matrices, generated between the average time course for each seed and each voxel in the brain.

**Results:** Based on SPC, we distinguished improvers—i.e., participants who showed impaired performance at baseline and reached the EMT psychophysical threshold during CCT—from maintainers—i.e., those who showed intact EMT performance at baseline and sustained the EMT psychophysical threshold throughout CCT. Compared to maintainers, improvers showed an increase of rsFC at rest between PCC and left superior and medial frontal regions and the cerebellum. Compared to improvers, maintainers showed increased rsFC at baseline between PCC and superior temporal and insular regions bilaterally.

**Conclusions:** In ROP patients with an increase of connectivity at rest in the default mode network, social CCT is still able to induce sensory processing changes that however do not translate into social cognitive gains. Future studies should investigate if impairments in short-term synaptic plasticity are responsible for this lack of response and can be remediated by pharmacological augmentation during CCT.

## Introduction

Cognitive system dysfunction represents a significant risk factor for ROP (recent-onset psychosis) (1, 2) and a poor prognostic indicator (3). Functional outcome in ROP is predicted by the level of cognitive impairments and in particular by impairments in social cognition (4). Therefore, cognitive dysfunction and underlying neural system inefficiency have become primary targets for preemptive experimental interventions in ROP, including computerized cognitive training (CCT) (5).

According to its hypothesized mechanism of action, CCT systematically improves cognitive–perceptual abilities by means of “drill and practice” exercises that induce neuroplastic changes in distributed neural systems, ultimately resulting in more efficient detection, processing, and resolution of sensory stimuli (6, 7).

In patients with ROP, a CCT program targeting the auditory system was found to significantly improve several domains of cognition, as well as early structural and dynamic imaging responses in auditory and prefrontal cortices (8–10).

More recently, the principles of CCT have been adapted to the processing of socially relevant information. A social CCT program was found to induce positive effects on cognitive performance (11), and to restore neural activity in patients with psychosis (12).

Findings from studies on neural underpinnings of auditory CCT indicate that the high heterogeneity of response is likely due to variable engagement of the targeted neural system (9, 13, 14). An indirect measure of target engagement is the degree of subject-specific, intrinsic sensory learning behavior that can be observed within the training exercises. For example, a recent study found that the greater a patient's ability to reach a performance threshold in one of the most basic auditory exercises during CCT, the greater the degree of improvement in global cognition after training (15). This suggests that modeling patterns of sensory processing change (SPC) may offer valuable information about mechanisms of response to CCT.

Recent lines of evidence seem to suggest that the degree of SPC occurring during training may be explained by interindividual differences in the efficiency of the neural systems underlying information processing (16). For example, a recent study in ROP (17) showed that greater deficits in mismatch negativity, an event-related potential elicited pre-attentively that indexes the efficiency of prefrontal-temporal neural systems underlying auditory processing, predicted greater improvements after auditory CCT.

While mechanisms of action of and response to auditory CCT have been well-studied, less is known about social CCT. In particular, no studies to date have modeled patterns of SPC as proxies of target engagement during social CCT. Some studies have characterized the neuroplastic changes following social cognitive interventions in schizophrenia (18), Yet, no studies have investigated whether the neural systems subserving social information processing vary among ROP patients with different patterns of SPC during training.

Resting-state functional connectivity fMRI (rsFC) is a neuroimaging technique that is ideally suited to study the neural correlates of engagement with social CCT in ROP for several reasons. First, it is well-understood that two key regions of the default mode network (DMN) (19–21), namely, the posterior cingulate cortex (PCC) and the medial prefrontal cortex (mPFC) (22), are implicated in many aspects of social cognition including emotion processing, emotion regulation, mentalizing, and perspective taking and are activated during social cognition tasks (23, 24). Second, while mPFC and PPC may not be specific for emotional processing; they both represent hubs involved in multiple functional networks (25) and their high centrality makes them susceptible to disconnection and dysfunction, which are of special interest in psychosis. Third, a growing body of work using rs-fcMRI suggests that DMN suppression may be compromised in schizophrenia during performance of cognitively demanding tasks, not as a result of suboptimal task engagement, and contribute to cognitive impairment observed in this illness (26). Importantly, the central executive network (CEN) that is anti-correlated with the DMN and comprises the dorsolateral prefrontal cortex and posterior parietal cortex is engaged during cognitively demanding tasks requiring attention (27). Fourth, CCT was shown to induce in patients with schizophrenia less functional connectivity loss between the PCC and the prefrontal cortex (PFC) (28). With mPFC and PCC serving as a backbone of social cognitive abilities, studying connectivity of these regions to other cortical and midbrain structures may shed light on the patterns of SPC that underlie target engagement and ultimately response to social CCT.

In the current study, conducted in a sample of ROP patients that underwent 10 h of social CCT, we first used performance data from the most basic social CCT exercise to model SPC, with the goal of identifying patterns of target engagement. Next, we investigated whether patients with distinct patterns of SPC showed differential response to treatment, as indexed by gains on a well-validated neuropsychological test for social cognition and, more specifically, emotion recognition. Finally, we used data from a baseline rsFC analysis performed using PFC and PCC as seeds to examine whether patients with distinct profiles of target engagement are characterized by unique rsFC.

## Methods

### Participants

Study participants were recruited from the Early Detection and Intervention Center at the Department of Psychiatry and Psychotherapy of the Ludwig-Maximilians-University (LMU) in Munich, Germany. In the context of a double-blind, randomized controlled trial comparing social CCT to treatment as usual (ClinicalTrials.gov Identifier: NCT03962426), here we only analyzed data from participants randomized to social CCT and completed the intervention (*n* = 26) (Supplementary Materials, Figure 1).

ROP participants had to meet criteria for an affective or non-affective psychotic episode as established by the Structured Clinical Interview for DSM-IV-TR (SCID) (29) or transition criteria defined by Yung et al. (28) and be within 3 months of onset of first treatment with antipsychotic medication. Specific ROP exclusion criteria were (1) onset of psychosis *spectrum diagnose* exceeding the past 24 months and antipsychotic medication exceeding 90 days (cumulative in the past 24 months) and (2) daily dose rate at or above minimum dosage of the “First Episode Psychosis” range of German Society for Psychiatry, Psychotherapy, and Nervous Diseases (DGPPN) S2 guidelines, with equivalency to 5 mg Olanzapine.

Exclusion criteria for ROP participants were (1) history of neurological disease, head trauma with loss of consciousness (>5 min), alcoholism or polytoxicomania; (2) insufficient intellectual capacity according to Wechsler Intelligence Scale for Adults [WAIS; (30)] IQ < 70; (3) violation of MRI safety requirements; (4) insufficient German language proficiency; and (5) prior cognitive training within the past 3 years [*further details can be found in* Haas (31)].

### Procedures

All participants provided written informed consent prior to study inclusion. All procedures performed in this study were in accordance with the ethical standards of the Local Research Ethics Committee of the LMU and with the 1964 Helsinki Declaration and its later amendments or comparable ethical standards.

After baseline clinical, neuropsychological and neuroimaging assessments were conducted (see below), participants randomized to social CCT were asked to complete 10 h of training over the course of 5 weeks (30 min per session, 4–5 days per week). The first three training sessions took place at the *Department of Psychiatry and Psychotherapy* of LMU. Next, participants had the option to attend group-based training sessions in the clinic or to train from home. Fifteen participants completed the remaining training sessions at the clinic, whereas 11 trained from home. While in the trial, participants received early intervention services by providers or clinic personnel not involved in the study (e.g., individual, group, and family therapy, case management, psychosocial rehabilitation, psychosocial education, psychiatric services, peer support services, and supportive employment and education services). Clinical and neuropsychological assessments were repeated after training completion. Demographic characteristics are presented in Table 1.

**Table 1:** Baseline demographic information of the intervention CCT sample.

	**Maintainers (*N* = 14)**	**Improvers (*N*= 12)**	***T*/**χ^2^****	***P*-value**
Number of female (%)	8 (57.14%)	3 (25.00%)	2.74	0.098
Age (*SD*)	27.46 (5.84)	26.10 (7.00)	0.54	0.594
Years education (*SD*)	14.96 (2.71)	15.79 (4.73)	−0.56	0.582
Premorbid IQ (*SD*)	97.14 (16.02)	100.83 (13.62)	−0.63	0.537
Handedness	-	-	2.20	0.333
Right (%)	9	11	-	-
Mixed (%)	2	0	-	-
Left (%)	1	1	-	-
Diagnosis	-	-	6.55	0.477
Schizophrenia (%)	4 (28.57 %)	4 (33.33 %)	-	-
Schizoaffective disorder (%)	1 (7.14 %)	-	-	-
Schizophreniform disorder (%)	1 (7.14 %)	2 (16.67 %)	-	-
Brief psychotic disorder (%)	3 (21.43 %)	3 (25.00 %)	-	-
Delusional disorder (%)	1 (7.14 %)	2 (16.67 %)	-	-
Psychotic disorder NOS (%)	1 (7.14%)	-	-	-
MDD with psychotic symptoms (%)	3 (21.43 %)	-	-	-
Substance-induced psychotic disorder (%)	-	1 (8.33 %)	-	-
**Medication at baseline (*****N*** **=** **39)**				
CPZ equivalent (*SD*)	142.68 (162.49)	278.44 (258.96)	−1.63	0.117
Days between assessments	51.29 (13.12)	47.42 (8.99)	0.86	0.397
GAF past month	46.25 (13.86)	48.00 (16.87)	−0.29	0.774
**GF current**				
Role (*SD*)	4.57 (1.45)	4.25 (1.54)	0.55	0.590
Social (*SD*)	6.00 (1.30)	6.00 (0.95)	0.00	1.000
**PANSS**				
Total (*SD*)	66.07 (15.61)	69.83 (17.94)	−0.57	0.573
Positive (*SD*)	19.21 (6.12)	19.83 (5.88)	−0.26	0.796
Negative (*SD*)	13.43 (5.24)	15.83 (6.19)	−1.07	0.294
General (*SD*)	33.43 (9.10)	34.17 (9.11)	−0.21	0.839

### Cognitive Training Intervention

Social CCT consists of four computerized exercises which collectively target perception, attention, and memory in the social cognitive domains of visual affect perception and social cue perception (gazes and faces).

The training employs a carefully designed stimulus set that allows progressive training of speed and accuracy (11). The training program is structured in blocks: early blocks operate with more simple stimuli to optimize more fundamental processes such as speeded responses and only once they are consolidated; later blocks appear with more naturalistic properties of stimuli, which apply to real-world performance. The stimulus set uses emphasized (e.g., high contrast, temporally stretched) stimuli in early blocks to drive strong synchronized brain responses and progressively moves to increasingly difficult discriminations in later blocks with respect to (1) stimulus complexity; (2) number of response alternatives; and (3) stimulus and response presentation times. This ensures that the exercises become more or less challenging at exactly the appropriate rate for a specific individual's rate of learning.

Each block consists of 20–50 adaptive trials. Within each block, sophisticated adaptive tracking methods are employed to continuously adjust a single adaptive dimension of the task to capabilities of the participant. This adaptive process is based on a statistically optimal Bayesian approach that allows the exercise to rapidly adapt to an individual's performance level and maintain the difficulty of the stimulus sets at an optimal level for driving efficient learning. This adaptivity operates from trial to trial, locking an individual's performance to 75–80%.

Therefore, even if the length of a training session (30 min) and the number of exercises within a session is fixed, different individuals complete different amounts of blocks per exercise within a session. Descriptions for each exercise can be found in the Supplementary Materials, Table 1.

Two metrics are available for each exercise: (1) baseline performance—this is the score reached the first time a participant completed any given exercise; (2) best performance—this is the best score reached in a training exercise at any point throughout the intervention. Correct trials are rewarded with auditory feedback, points, and animations. Compliance is monitored by electronic data upload. Further details regarding the training are available (11).

### Target Engagement

While all four exercises target early social sensory processing, we chose to study the Emotion Matching Task (EMT) as a potential proxy for target engagement, given its ability to capture the processing of basic social information. In this speeded exercise, participants are shown a target face displaying an emotion and then asked to select from a set of other images which one displays the same facial expression. The exercise is designed to improve the ability to make implicit speeded decisions about facial emotions.

**Figure 1:**
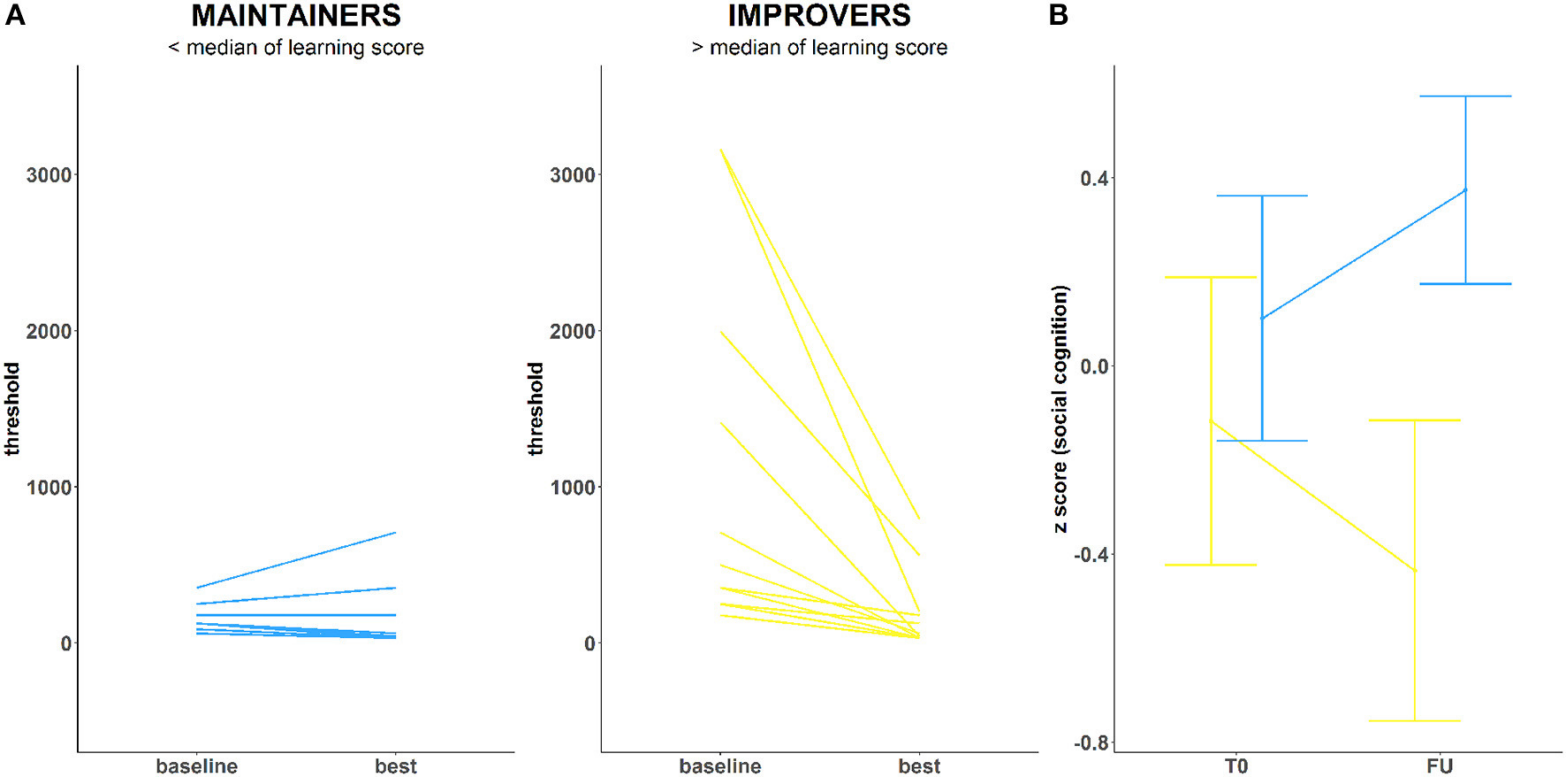
**(A)** Training performance in two ROP groups: Maintainers (left) and Improvers (right) **(B)** Emotion Recognition (DANVA) change over the course of training in both groups.

Twenty-four unique blocks of EMT are available throughout the training. To model SPC, we chose one that (1) was completed at least once by all participants and (2) provided the largest amount of block repetitions per participant. SPC on this exercise was calculated by dividing the difference between subject-specific best and baseline performance within that block, by the standard deviation of baseline performance for that block across all study participants. The larger score for best-baseline/SD (baseline) occurs for those with a greater delta who did not start with the training too well but managed to improve. A smaller score for best-baseline/SD(baseline) characterizes instead those with a smaller delta driven by an exceptionally good baseline performance. *Due to the social CCT, they all reach the same goal*. Based on the median split of such improvements (Figure 1A), participants were dichotomized into “improvers” (*n* = 12) and “maintainers” (*n* = 14). Improvers are participants who showed impaired performance at baseline and reached the psychophysical threshold for EMT (~31 ms) during training (high SPC). Maintainers are participants who showed intact psychophysical threshold for EMT at baseline (~31 ms) and sustained it throughout the training experience (low SPC).

### Clinical and Neuropsychological Assessments

The following tools were administered before and after social CCT. The Positive and Negative Syndrome Scale (PANSS) was administered to assess presence and severity of symptoms (32, 33). Real-world functioning was assessed using the Global Assessment of Functioning (GAF) Disability and Impairment Scale of the DSM-IV (34). A cross-domain neuropsychological test battery comprising of 9 tests were administered to patients in the intervention sample at baseline (T0) and follow-up (FU) in a fixed order. After winsorizing and checking for outliers, tests were z-score transformed based on the study sample to closely reflect cognitive domains based on the Measurement and Treatment Research to Improve Cognition in Schizophrenia (MATRICS) (35) recommended procedures (Supplementary Materials, Table 2). In particular, social cognitive abilities were assessed using the emotion recognition *task* Diagnostic Analysis of Non-verbal Accuracy-2 (DANVA-2) (36) test.

### Imaging Preprocessing Procedure

Both structural magnetic resonance imaging (sMRI) and resting-state fMRI (rsfMRI) were acquired from all participants on a 3-Tesla Philips Ingenia scanner with a 32-channel radio-frequency coil at the Radiology Department in the university clinic of the LMU, in Munich, Germany.

The CAT12 preprocessing procedure of the sMRI T1 images is described in the Supplementary Materials. rsfMRI preprocessing was divided into two main processes: core and denoising steps based on Patel et al. (37). Core preprocessing consisted of the following and were performed using Statistical Parametric Mapping, version 12 (SPM12) (https://www.fil.ion.ucl.ac.uk/spm/software/spm12/) version 6685. After initially discarding the first 8 volumes, the remaining 192 images were slice-time corrected and then unwarped and realigned to the first volume for head-motion correction. The time course of head motion was obtained by estimating the translations in each direction and the rotations in angular motion about each axis for each volume. Next, framewise displacement (FD) was calculated for each subject (ref). FD for the first volume of a run is by convention zero. Subjects with >38.5% of volumes with mean FD of > 0.50 mm were excluded from further analyses (38).

Affine coregistration of images to structural images followed and were then resliced using 4th-degree B-Spline interpolation. The standard CAT12 template was converted from DARTEL space to MNI space using SPM12's population to International Consortium for Brain Mapping 152 registration procedure. The resulting image was used as a deformation field to normalize all coregistered images to MNI space. Next, gray matter (GM), white matter (WM), and CSF masks were created using an image calculator procedure within SPM12 using thresholds of 0.20, 0.20, and 0.50, respectively. Subsequently, Friston 24 motion parameters (39) including six motion parameters, six temporal derivatives, six quadratic terms, and six quadratic expressions of the derivatives of motion estimates were derived. Then, mean individual signal estimates with variance regressed out from WM and CSF were generated. Finally, functional volumes were masked using the GM mask to limit space and spatial smoothing using a Gaussian kernel of 6-mm full width at half-maximum was applied.

Denoising methods consisted of motion correction using time series despiking (Wavelet Despike) with the BrainWavelet Toolbox (http://www.brainwavelet.org/). The following steps were done using the Resting-State fMRI Data Analysis Toolkit (REST version 1.8; http://www.restfmri.net/) (40). Confound signal regression of the Friston 24 motion parameters, and residuals of WM and CSF was applied. Finally, the images underwent background filtering and temporal band-pass filtering (0.01–0.08 Hz) was performed to reduce the effects of low-frequency drift and high-frequency noise [further details can be found in Haas (31)].

### Statistical Analysis of Behavioral Data

We screened all variables for normality after winsorizing outlying values (>3 standard deviations from the mean). We chose to compare improvers (*n* = 12) and maintainers (*n* = 14) based on the median split of their improvement on the EMT. Independent-sample *t*-tests were used to explore baseline differences in demographic variables, hours of training, medications, and days between assessments. Fisher's chi-square tests were used to explore group difference for categorical variables (i.e., gender, diagnosis, handedness). Between-group differences in clinical and neuropsychological outcomes were studied using an analysis of covariance (ANCOVA), with follow-up scores as a dependent variable, baseline performance as a covariate, and condition (improvers, maintainers) as a between-subject factor. Significance levels were defined at *p* = 0.05 with false discovery rate (FDR) correction for multiple comparisons (41). All analyses were conducted using Jamovi 22 (https://www.jamovi.org/).

### Statistical Analysis of Neuroimaging Data

Based on our strong a priori hypothesis, a seed-based rsFC analysis was performed using mPFC and PCC (42). BOLD time series were first extracted from a 3-millimeter (mm)-radius sphere centered at the coordinates (-7,49,18) for the mPFC and (-7,-52, 26) for PCC. Left-lateralized mPFC and PCC were used since several neuroimaging studies suggest left lateralization of the default mode network (43, 44). Using the Resting-State fMRI Data Analysis Toolkit (REST version 1.8) (40), a correlation map was produced by computing the Pearson correlation coefficients between the average time course that was extracted for each seed and each voxel in the whole brain for every subject. Finally, correlation coefficients were converted to z-values using Fisher's r-to-z transform for each subject to improve normality and allow for parametric testing. According to factorial design, individual z-maps were entered into independent sample *t*-tests which were conducted to compare connectivity between the respective seed and the rest of the brain voxels between improvers and maintainers.

To sensitize our neuroanatomical analysis both for large focal and subtle, spatially contiguous effects, we used Threshold-Free Cluster Enhancement (TFCE) as implemented in the SPM TFCE toolbox (45). We performed *N* = 2,000 permutations of each previously generated contrast in SPM. Statistically significant effects in the TFCE maps were defined at *P* < 0.05, corrected for multiple comparisons using the false-discovery error rate (FDE) (41).

## Results

### Behavioral Results

At baseline, there were no significant differences between improvers and maintainers in demographic characteristics, symptom severity, functioning, number of days between assessments, training intensity, or antipsychotic medication (*p* > 0.05) (Supplementary Materials, Table 3). We observed a marginally significant between-group effect on the emotion recognition FU scores (*F* = 4.45, *p* = 0.046), while controlling for T0 performance (*F* = 4.08, *p* = 0.055). Maintainers showed significant improvements in DANVA-2 scores, with small to moderate effect size (Cohen's *d* = 0.33) (Figure 1B). Conversely, improvers showed significant deterioration of DANVA-2 performance, with a negative effect size in a small to moderate range (Cohen's *d* = −0.29). No significant between-group differences were found either for other cognitive domains (*p* > 0.05), symptom severity (*p* > 0.05), or functioning (*p* > 0.05).

### Neuroimaging Results

**Figure 2:**
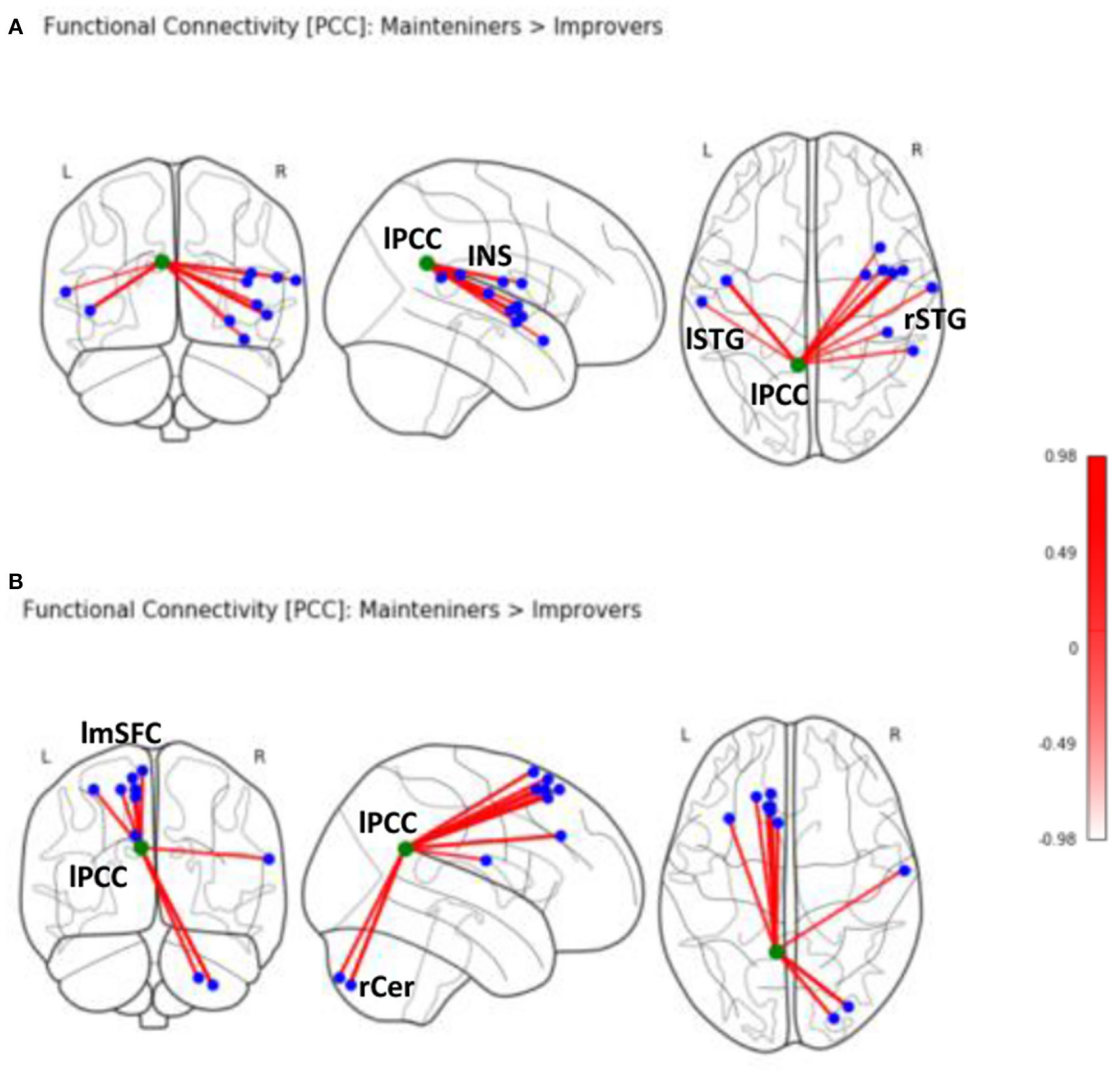
Seed-based rsFC of posterior cingulate (PCC) on MNI schematic template (https://nilearn.github.io) in **(A)** Maintainers > Improvers (INS, insula; lSTG, left superior temporal gyrus; rSTG, right superior temporal gyrus). **(B)** Seed-based rsFC of PCC in Improvers > Maintainers (rCer, right cerebellum; lmSFC, left medial superior frontal cortex).

Second-level analyses revealed a number of significant differences in whole-brain rsFC of left PCC between improver and maintainer patients that underwent SCT (Table 2). Compared to maintainers, improvers showed at rest an increase in connectivity between left PCC and left superior medial frontal lobe (including supplementary motor area, frontal inferior lobe, triangularis lobe, and left thalamus (Figure 2), as well as an increase in connectivity between left PCC and right postcentral gyrus. Additionally, PCC connectivity to the right portion of the cerebellum was increased in improvers as compared to maintainers. Compared to improvers, maintainers showed increased connectivity between the left PCC and superior temporal pole (STP) bilaterally, right insula, and right putamen (Supplementary Materials, Table 3) (for more detail see Supplementary Materials, Figure 2). No differences in left mPFC connectivity between improvers and maintainers remained significant after FDR correction.

**Table 2:** Seed based PCC (-7,-52,26) rsFC in the two ROP groups.

	**Region**	**Coordinates**			**KE**	**Side**	**TFCE scores**
		**X**	**Y**	**Z**			
Maintainers > Improvers	Insula	50	0	−3	106	R	976.98
	Insula	−46	−6	0	64	L	968.93
	Superior temporal gyrus	56	−44	18	32	R	597.77
	Postcentral gyrus	66	−10	16	16	R	590.62
	Insula	39	0	15	15	R	602.75
	Insula	38	12	−16	14	R	583.51
	Putamen	30	−3	−6	13	R	534.37
	Rolandic oper.	42	−34	20	5	R	534.13
	Insula	45	−2	3	2	R	765.45
	Superior temporal	−60	−18	10	2	L	524.62
Improvers > Maintainers	Cerebellum crus	32	−82	−48	142	R	740.47
	Supplementary motor	−6	18	68	47	L	672.24
	Frontal Supp. Medial	−10	26	54	8	L	703.85
	Postcentral gyrus	63	−8	20	13	R	522.36
	Superior frontal gyrus	−12	26	64	8	L	644.18
	Superior frontal medial	−10	33	33	6	L	683.35
	Cerebellum	24	−88	−44	4	R	651.28
	Middle frontal gyrus	−33	20	58	4	L	588.05
	Supp.motor area	−10	24	58	2	L	651.79
	Superior frontal	−18	32	58	2	L	610.53

*All scores significant (p < 0.05) after false discovery rate (FDR) correction for multiple comparisons over the whole brain. X-Y-Z coordinates are according to the Montreal Neurological Institute (MNI) coordinate system*.

## Discussion

To our knowledge, the current study is the first to study and model target engagement during social CCT in a sample of ROP patients. Similarly to what has been done for auditory CCT (15, 46), we chose performance change on an EMT exercise that trains early social sensory processing. An analysis of subject-specific learning curves identified two classes of target engagement, showing different improvements in the social cognitive task and different rsFC of the left PCC with fronto-temporal, insular, and cerebellar brain regions. In particular, we identified a subgroup of participants who initially presented with social sensory processing impairments. Upon exposure to social CCT, this subgroup showed significant improvements in sensory processing and we labeled these ROP participants as “improvers.” The other subgroup of ROP participants that we labeled “maintainers” initially presented with unimpaired social sensory processing and maintained peak performance throughout the training at the optimal psychophysical level.

The analysis of behavioral data from a well-validated social cognitive task indicated that these two profiles of target engagement are associated with distinct responses to social CCT. While maintainers showed significant improvements in emotion recognition, with a small-medium effect size, improvers showed a deterioration of emotion recognition after CCT, with a negative effect size in the same range. This suggests that there is a subgroup of ROP participants for which improvements in the training exercises do not translate into gains in untrained cognitive measures. This is in line with results from two RCTs of auditory CCT in chronic psychosis that found overall improvement on the training exercises but no transfer of these gains to untrained cognitive outcomes despite endurable training regime (47, 48). Conversely, our ROP participants with more cognitive reserve—as indexed by fast sensory processing at baseline—showed greater transfer effects to the domain of emotion recognition, replicating findings from two studies of social CCT conducted in chronic psychosis (11, 49).

Armed with this information, we sought to investigate whether distinct patterns of baseline rsFC could explain why improvers and maintainers show such divergent profiles of target engagement and response to social CCT. Compared to improvers, maintainers showed evidence at rest of increased connectivity between the left PCC and several areas involved in emotional and social processing, including superior temporal pole (STP), insula, and putamen. Our findings support the notion that insula hosts the mechanism of emotion discrimination, of negative emotion in particular (50), alongside the basal ganglia and amygdala. Moreover, the connectivity of the PCC to upper portion of STP provides a strong functional integration platform for the facial affect recognition (51).

Improvers, conversely, showed at rest increased connectivity between the left PCC and several frontal regions functionally correlated with the DMN (52). In this subgroup of participants, the left PCC also showed increased connectivity with parts of fronto-parietal CEN. The medial area of superior frontal lobe, including the supplementary motor area, is involved in cognitive control (22). Though the thalamus is not a part of CEN, it is a key region in integrating neural activity from widespread neocortical inputs and outputs, particularly in tasks requiring high degree of attentional control (53). In this regard, cognitive gains induced by auditory CCT in a sample of ROP patients were found to be associated with structural neuroplasticity in the thalamus (10). Taken together, these findings show compromised suppression within the DMN network, as well as increased rsFC of the PCC to CEN nodes and postcentral gyrus (54) at rest in improvers vs. maintainers.

We suggest that improvers express, stronger than maintainers, the psychosis endophenotype that is characterized by increased connectivity of DMN (55). The distinct lack of DMN suppression in our ROP improvers subsample replicates a large body of studies that observed increased connectivity at rest in schizophrenia within the DMN hubs and between DMN hubs and extra-DMN areas (55). In this context, deficits in DMN suppression likely exemplify additional forms of cortical circuit dysfunction associated with ROP that compromise task-relevant signal processing, adding to task-related cortical activation deficits that underlie cognitive impairments. Moreover, the involvement of CEN parts, including superior frontal regions, points to difficulties in smooth interplay between task-negative and task-positive activity (56) that enables optimal cognitive functioning. As a matter of fact, while “improvers” actively engage with the CCT target and show sensory processing change, such changes do not translate into cognitive gains after training. We suggest that the cortical circuit dysfunction typical of this endophenotype could originate from impairments of short-term synaptic plasticity mechanisms in the service of sensory learning (57–59). Accordingly, ROP individuals with greater impairments in synaptic plasticity would only be able to generate, but not to sustain, successful learning in response to the training trials, and this inability, in turn, would manifest as lack of cognitive gains after CCT. For these individuals, we believe that the augmentation of CCT with pharmacological agents targeting and remediating impairments in synaptic plasticity has potential to generate durable cognitive improvements (60).

### Limitations

The current study has several limitations. Notably, despite PCC being a key component of the DMN, this study was not aiming at studying networks, but seed-to-brain voxel connectivity. Additionally, mPFC as a seed did not show significant results in our analysis. Though we interpret our results in the framework of DMN and CEN, they are restricted to rsFC within only certain parts of these networks. Further studies employing independent component analysis would be necessary to extend our claims. Next, only 10 h of social CCT were delivered over the course of 5 weeks. This reflects the need to incorporate the experimental intervention into the intensive treatment package that is routinely offered in our clinical setting. We cannot exclude the possibility that improvers would have shown a significant improvement in outcomes after longer or more diversified CCT protocols (61). However, the most prominent changes in sensory processing, tightly linked to heterogeneity in neuroplastic response to CCT, have been shown in the early stages of the training, whereas it has been suggested that after 20 h of CCT sensory processing undergoes less change (15).

Further, our study was lacking a group of healthy volunteers that would have provided a stronger base to discuss aberrant rsFC in ROP patients. Finally, we acknowledge that an individual approach in defining seeds may reduce spatial variability and increase accuracy of rsFC analysis (62); future studies of social CCT with larger sample sizes should confirm the existence of the two classes of indicated in this study.

## Conclusion and Future Directions

Our findings reveal that interindividual differences in PCC connectivity to fronto-temporal-insular brain regions may result in different patterns of sensory processing change upon exposure to social CCT, which can significantly influence treatment response. We believe that these lines of investigation are critical for two reasons. First, once we identify behavioral proxies for sensory processing patterns mediating treatment response, it becomes possible to determine treatment uptake for a given individual very early in the course of social CCT. This can ultimately translate in the implementation of fast-fail approaches that promote the maximization of benefits for individuals sensitive to the intervention, thus enhancing cost-effectiveness. Second, once the subgroup of patients showing efficacious neural target engagement is identified by means of these behavioral proxies, we can truly begin to study the neuroplastic effects directly induced by training. This will promote a characterization of the mechanisms of action of CCT, paving the way for a data-driven optimization and refinement of this treatment.

## Data Availability Statement

The raw data supporting the conclusions of this article will be made available by the authors, without undue reservation.

## Ethics Statement

The studies involving human participants were reviewed and approved by Local Research Ethics Committee of the Ludwig-Maximilians-University. The patients/participants provided their written informed consent to participate in this study.

## Author Contributions

LK-I and BB conceptualized the paper. LK-I and NK oversaw data collection and project development. LK-I was responsible for statistical analysis. BB and LK-I drafted the manuscript and provided data interpretation. JW and SH assisted in statistical analysis and data interpretation. SH, JW, and RS assisted in data collection and data entry. LA, SH, and AR were involved in developing neuroimaging pipeline. MP was in charge of developing scanning protocols. All authors revised and agreed upon the final version of the manuscript.

## Conflict of Interest

BB is Senior Scientist at Posit Science, a company that produces cognitive training and assessment software. The training programs described in this study were provided for research purposes free of charge by Posit Science. The remaining authors declare that the research was conducted in the absence of any commercial or financial relationships that could be construed as a potential conflict of interest.
