# Supplementary material for: Modeling Social Sensory Processing During Social Computerized Cognitive Training for Psychosis Spectrum: The Resting-State Approach
Source: Front Psychiatry. 2020 Nov 19;11:554475. doi: 10.3389/fpsyt.2020.554475 (PMC7716799; doi:10.3389/fpsyt.2020.554475)
Supplement: Supplementary file 1 [file Data_Sheet_1.docx]

**MRI processing pipeline of structural data**

The manual of the CAT12 toolbox, version r>1200 (<http://www.neuro.uni-jena.de/cat12/CAT12-Manual.pdf>) details the processing steps applied to the structural images. These steps consist of:

1. A 1^st^ denoising step based on Spatially Adaptive Non-Local Means (SANLM) filtering)(29).
2. An Adaptive Maximum A Posteriori (AMAP) segmentation technique, which models local variations of intensity distributions as slowly varying spatial functions and thus achieves a homogeneous segmentation across cortical and subcortical structures(30).
3. A 2^nd^ denoising step using Markov Random Field approach which incorporates spatial prior information of adjacent voxels into the segmentation estimation generated by AMAP(30).
4. A Local Adaptive Segmentation (LAS) step, which adjusts the images for white matter (WM) inhomogeneities and varying gray matter (GM) intensities caused by differing iron content in e.g. cortical and subcortical structures. The LAS step is carried out before the final AMAP segmentation.
5. A Partial Volume Segmentation algorithm that is capable of modeling tissues with intensities between GM and WM, as well as GM and cerebrospinal fluid (CSF) and is applied to the AMAP-generated tissue segments.
6. (6) A high-dimensional DARTEL registration of the image to a MNI-template generated from the MRI data of 555 healthy controls in the IXI database (http://www.braindevelopment.org). The registered GM images were multiplied with the Jacobian determinants obtained during registration to produce GM volume (GMV) maps.

The Quality Assurance framework of CAT12 was used to empirically check the quality of the GMV maps. By computing the correlation of each image to all other images, taking the original and independent sample separately, we removed two images whose correlation exceeded 2 standard deviations from the sample mean due to MRI artifacts.

**Table 1.** Description of the social cognitive training exercises provided by Posit Science, Inc. (SocialVille) used for the intervention in the order of administration

| Exercise | Trials per iteration^*^ | Description | Target |
| --- | --- | --- | --- |
| Recognition | 20 | A speeded face matching task: Select the correct target face from an array of faces | Improve processing of facial features. |
| Emotion Matching Task | 20 | A speeded facial emotion matching task: Select the face showing the same facial expression as the target face | Improve the ability to make implicit speeded decisions about facial emotion features. |
| Gaze Match | 40 | A speeded gaze matching task: Match gaze direction of target face | Improve processing speed for accurate identification of eye gaze direction. |
| Face Poke | 60 | A CPT task with facial expressions: Withhold response for neutral expressions (10 % of trials), respond quickly to emotional faces (90 %) | Improve the brain's ability to distinguish between emotionally expressive faces and neutral faces. |

**Table 2.** The cognitive domains assessed and a description of their respective tests.

| Neurocognitive domain | Cognitive test | Description of tests |
| --- | --- | --- |
| *Social cognition/Emotion Recognition* | Diagnostic Analysis of Nonverbal Accuracy-2 | A test of social cognition measuring the ability to read nonverbal social information. |
| Speed of processing | Trail Making Test (TMT): Part A | A test of visual scanning and visuomotor tracking |
|  | Verbal Fluency: semantic | A verbal index of speed of processing |
|  | Wechsler Adult Intelligence Scale, 3rd ed., digit symbol coding task | A measure of visuomotor speed |
| Working memory | Wechsler Memory Scale, 3rd ed., spatial span subtest | A measure of nonverbal working memory |
| Verbal learning | Rey Auditory Verbal Learning Test (RAVLT) | A list of 15 words presented 5 times, which must be recalled from memory |
| Attention | Continuous Performance Task - Identical Pairs (CPT-IP) | A measure of attention and vigilance |
| Executive functioning | Trail Making Test (TMT): Part A2 | A test measuring cognitive flexibility and set shifting ability |
|  | Verbal Fluency: phonetic | A verbal index of executive ability |
| Global cognition | Composite across all cognitive measures included above (average z-score) | A global measure of cognitive functioning |

**Table 3.** Cognitive measures, symptom ratings, and functional outcomes at baseline and follow-up of maintainers and improvers

|  | Maintainers F2F  (N = 14) | | Improvers F2F  (N = 12) | | Main Effect of Time *F* (*P*) | Interaction (Group x Time)  *F* (*P*) |
| --- | --- | --- | --- | --- | --- | --- |
|  | T0  (SD) | FU (SD) | T0  (SD) | FU  (SD) |  |  |
| **Cognition** |  |  |  |  |  |  |
| Global cognition | 0.14 (0.55) | 0.19 (0.67) | -0.16 (0.71) | -0.22 (0.49) | 0.002  (0.96) | 0.39  (0.54) |
| *Social cognition/Emotion Recognition* | 0.10 (0.98) | 0.37  (0.75) | -0.12 (1.06) | -0.44 (1.11) | 0.01  (0.92) | 1.94  (0.18) |
| Speed of processing | 0.03 (0.83) | 0.08 (0.94) | -0.03 (0.77) | -0.10 (0.58) | 0.002  (0.97) | 0.32  (0.58) |
| Working memory | 0.04 (0.95) | 0.08 (0.97) | -0.04 (0.76) | -0.01  (0.73) | <0.001  (0.99) | 0.04  (0.84) |
| Verbal Learning | 0.37 (0.72) | 0.27 (1.09) | -0.43 (1.14) | -0.31 (0.82) | 0.003  (0.96) | 0.53  (0.47) |
| Attention | 0.17  (1.13) | 0.20 (0.88) | -0.20 (0.83) | -0.23 (1.12) | <0.001  (0.99) | 0.03  (0.86) |
| **Functional Outcome** |  |  |  |  |  |  |
| GAF global rating past month | 46.25 (13.86) | 58.82 (13.54) | 48.00 (16.87) | 57.83 (8.92) | 11.42 (0.002)** | 0.17  (0.68) |
| Global Functioning - Role | 4.57 (1.45) | 5.93 (1.49) | 4.25 (1.55) | 5.08 (1.38) | 14.62  (<0.001)*** | 0.84  (0.37) |
| Global Functioning - Social | 6.00 (1.30) | 6.71 (1.27) | 6.00 (0.95) | 6.42 (1.00) | 7.14  (0.013)* | 0.50  (0.49) |
| **Symptoms** |  |  |  |  |  |  |
| PANSS total | 66.07 (15.61) | 43.79 (16.72) | 69.83 (17.94) | 44.42 (12.75) | 30.98 (<0.001)*** | 0.13  (0.72) |
| PANSS positive | 19.21 (6.12) | 10.29 (4.25) | 19.83 (5.88) | 10.50 (3.12) | 48.26 (<0.001)*** | 0.02  (0.879) |
| PANSS negative | 13.43 (5.24) | 10.29 (4.91) | 15.83 (6.19) | 11.33 (4.25) | 9.31 (0.005)** | 0.29  (0.59) |
| PANSS general psychopathology | 33.43 (9.10) | 23.21 (8.79) | 34.17 (9.11) | 22.58 (7.00) | 22.68 (<0.001)*** | 0.09  (0.77) |

Total Enrolled: 68

Excluded During Baseline: 5

Total Randomized: 63

Total randomized: 30

Completers: 27

Non-Completers: 3

Total randomized: 33

Completers: 28

Non-Completers: 5

**CCT Group**

**TAU Group**

Outliers >3 cognitive domains: 1

Final Sample: 26

Incompatible MRI parameters: 1

Outliers >3 cognitive domains: 1

Final Sample: 26

**Figure 1. Flowchart of the study sample**


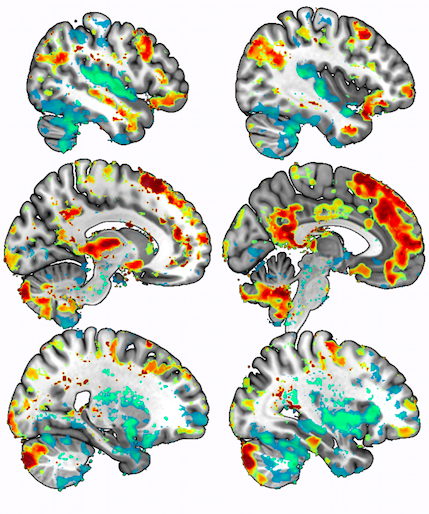


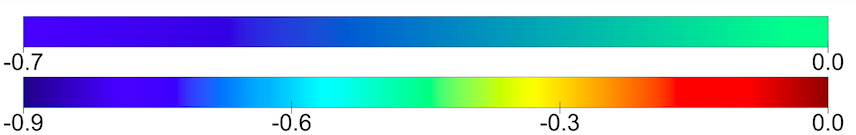


*Figure 2. rsFC of PCC in maintainers > improvers (cool color bar) and improvers> maintainers (warm color bar) on the MNI template (MRIcro GL* [*https://www.nitrc.org/projects/mricrogl*](https://www.nitrc.org/projects/mricrogl)*)*

**References**

1. Manjón JV, Carbonell-Caballero J, Lull JJ, García-Martí G, Martí-Bonmatí L, Robles M. MRI denoising using non-local means. Med Image Anal. 2008 Aug;12(4):514–23.

2. Rajapakse JC, Giedd JN, Rapoport JL. Statistical approach to segmentation of single-channel cerebral MR images. IEEE Trans Med Imaging. 1997 Apr;16(2):176–86.
